# Special issue on ‘Shifting Borders of European (In)Securities: Human Security, Border (In)Security and Mobility in Security’﻿

**DOI:** 10.1057/s41311-022-00375-y

**Published:** 2022-02-26

**Authors:** Stefania Panebianco, Benjamin Tallis

**Affiliations:** 1 University of Catania, Catania, Italy; 2 Hertie School, Berlin, Germany; 3 Institute of International Relations Prague, Nerudova 257, 118 00 Prague, Czech Republic

**Keywords:** EU, Borders, Migration, Human security, Mobility, Insecurity

## Abstract

Over the past decade, the European Union (EU) has faced a severe migration crisis that brought to the fore the issues of borders and security—including security at borders and the borders of security. This article introduces the special issue ‘Shifting Borders of European (In)Securities: Human Security, Border (In)Security and Mobility in Security’ going beyond the traditional dichotomy vision of borders, namely inclusion versus exclusion (Panebianco 2016). It sets the scene to examine the proliferating insecurities at and in reference to EU borders, suggesting a migrant-centred understanding of human security and mobility and its complex relations to—and tensions with—more traditional conceptions of border security. In particular, this introductory paper opens up the possibility of disentangling the complexity of (in)securities and (im)mobilities. Like the rest of the special issue, it shows that state security and human security are not mutually exclusive and can in fact be mutually reinforcing, even if this unfortunately remains the exception rather than the norm in practice. The paper (and the special issue) seeks to elaborate a more comprehensive and nuanced understanding of the challenges of shifting borders of European (in)securities, thus shedding light on complex migration phenomena and contributing to better understanding of these issues and what can be done about them in, but also beyond, academia.

The issue of security at (European) borders has long attracted the attention of scholars taking different disciplinary angles and focusing, variously, on EU politics (Schimmelfennig 2021), Critical Security Studies (C.A.S.E Collective 2006) or Border Studies (Johnson et al. 2011). Border crises can thus easily relaunch dormant scholarly debates or add fuel to the fire of ongoing discussions (Brambilla et al. 2015; Dell’Agnese and Amilhat Szary^ 2015^). The migration crisis of the 2010s, in particular the so-called 2015 refugee crisis, has brought insecurities over mobility to the fore. Indeed, plural, competing insecurities lie at the heart of how and why the European Union (EU) and its member states turned migration into a crisis (Pallister-Wilkins 2016; Attinà 2018), why they have been unable to react to ‘creeping crises’ such as migration (Landström and Ekengren 2021), why re-bordering was the first reaction to the refugee crisis (Kriesi et al. 2021) and how external bordering or re-bordering within the EU have come as a reaction to the COVID-19 pandemic (Schimmelfennig 2021).

The multiple insecurities in question include insecurity over the EU’s ability to manage its borders in ‘firm’ but ‘fair’ ways; insecurity over whether doing so violated the human security of migrants and undermined the EU’s status as a liberal actor; drastic human insecurity for distressingly large numbers of migrants; insecurity over the effects of increased inward migration on communities in EU Member States (EUMS); insecurity over whether, amid significant discord, the EU could maintain its model of governance, including in the Schengen zone, and its neighbourhoods; insecurity over the cohesiveness of the EU and over the future of the Union itself (Roos 2019); insecurity about the EU’s place in a changing world that will see increased migratory flows, which Europe needs demographically but remains wary of socially and politically.

But what to do with this proliferation and complexity of insecurity? How to map seemingly disparate phenomena in a coherent way without eliding their diversity? How to understand the effects as well as the underpinnings of these insecurities in relation to migration, including in order to challenge or confirm policies, practices and discourses—locally, nationally and/or at the European level? Answering these questions—and doing so in a way that contributes conceptually as well as empirically and methodologically—provides the rationale for this special issue of *International Politics* on ‘Shifting Borders of European (in)Securities’.

## Human security, increased migration and the EU’s ‘Mediterranean’ borders

This special issue takes a fresher, broader and deeper look at the migration crisis of the mid-2010s in the Mediterranean area through the lenses of ‘Human Security, Border (In)Security and Mobility in Security’ to better understand its implications for policies and polities, identities and order in Europe as well as, crucially, for people on the move. Understanding this intersection between (EU) borders and security requires that we move beyond the simplistic dual logic of exclusion (preventing people from arriving to Europe) *versus* inclusion (allowing for migrants to access Europe whether legally or illegally, permanently or temporarily) in order to disentangle the plural issues and tendencies involved (Panebianco 2016). We do so by focusing on the human dimension of (in)security and, throughout the volume, by relating this to other conceptions of (in)security and their related politics of (im)mobility.

Considering that ‘bordering practices are less and less the exclusive domain of the state and its agents’ (Johnson et al. 2011, 62), the daily work of making borders secure has become an increasingly sophisticated—and often opaque—task. Therefore, this collection seeks to go further in productively synthesising and juxtaposing literature works and approaches from cross and sub-fields of International Relations, Politics, European (Integration) Studies, Migration Studies, Security Studies, Border Studies and integrating insights from cognate disciplines such as Geography and Anthropology, to provide a deeper insight into the construction and consequences of the new borders of European insecurities. Opening with a new conceptualisation of bordering and its political implications (Tallis), it does so in a way that specifically and consistently situates these issues in the context of their implications for and in European Integration, European identities and order.

The volume asks, for example, how we can trace the lines of conflict, and of cooperation, of discriminations and prejudice (Bello) or complementarity between these different insecurities and the policies (Fontana, Kaunert and Leonard), the discourses (Herborth, Futak Campbell) and practices of security (Cusumano, Panebianco, Welfens) they give rise to (or stem from) as well as how they relate to mobilities and immobilities? To do so, we take a newly comprehensive approach: ontologically with regard to both the geographic and empirical scope of what constitutes bordering (Johnson et al. 2011, 67), We simultaneously consider the interplay of different kinds of security and insecurity (Huysmans 2006), insecure/risky mobility (McMahon and Sigona 2021) and immobility (Wolff et al. 2018), whether instantiated in discourse or practices of humanitarianism (Adler and Pouliot 2011; Adler-Nissen 2016; Panebianco 2019, 2020; Pallister-Wilkins 2020). We explore this across the range from human (in)security (Kaldor 2007) to border (in)security (Pallister-Wilkins 2015; 2018), from border security as practice (Côté-Boucher et al. 2014) to the politics of dwelling and moving within (and into) the Schengen zone (Walters 2006; Rajaram and Grundy Warr 2007; Börzel and Risse 2018). Moreover, the conceptual apparatus that frames the contributions to this collection brings coherence to this plurality, without eliding its diversity. It allows for zooming-out to the connections between bordering and geopolitical issues relating to European order and governance, as well as zooming-in to the effects of bordering on moving and dwelling people and their connections to identity and subjectivity (Albert et al. 2001; Johnson et al. 2011; Tallis, forthcoming).

This comprehensive approach shows how competing, often interlocking or overlapping conceptions of security and mobility (and related conceptions of insecurity and immobility) have produced new borderings within the EU as well as at its territorial frontiers and in its neighbourhood (Del Sarto 2016; Tallis, forthcoming; Zaiotti 2016). Nonetheless, it also shows that these new borderings have deep roots in long-standing social and political concerns as well as in EU and EUMS policies (e.g. Fisher Onar and Nicolaïdis 2013). Human Security, for example, has long been an element of official EU discourse and policy (Christou 2014), which brought a welcome decentring of the focus on states as both objects and providers of security, bringing in greater consideration the individuals’ security and the role of non-state actors, because ‘[c]itizens, entrepreneurs, and NGOs are active in constructing, shifting, or even erasing borders’. (Johnson et al. 2011, 67).

So too has border security (in various forms) and this also brought in new ‘referent objects’ of security (Waever 1995; Buzan et al. 1998): community security, political security, economic security, social and societal security (e.g. Guild 2009), and more recently climate security (e.g. Panebianco 2022) or health security. Moreover, mobility itself has emerged as a referent object of security: as a key underlying logic of the Schengen zone (a centrepiece of EU governance and order) and thus to be protected, but also of significant aspects of the EU’s neighbourhood policies (Isleyen 2018) which comes with a more ambiguous politics of protection (Tallis, forthcoming). Research that explores international migration governance via a decentring approach proposes a plural understanding drawing from the different dimensions of the analysis (in geopolitical, spatial or actor-perspective terms) (Triandafyllidou 2020). The conflicts between some aspects of these different forms of security have been noted elsewhere in particular examples, but this special issue goes further in systematically analysing them in relation to the EU’s migration crisis and with regard to their implications for European integration more generally (Zielonka 2017). Multiple and sometimes mutually exclusive understandings of mobility and immobility (which, like security and insecurity can be both imposed and/or desired) further muddy the waters: who gets to move to (and in particular ways) to particular places—and who gets to stay there or who is forced to stay elsewhere, even if they were forced to move in the first place—impact not only on (in)security but also on wider concerns relating to identity and order (e.g. Albert et al. 2001).

In order to better understand the effects of this complexity, on different groups of people as well as on the EU and its member states, this collection explores the ways in which these competing conceptions of (in)security and (im)mobility intersect to produce borders and borderings. These manifestations of bordering take place, in multiple forms, at ‘traditional’ locations at state frontiers (Fontana), but also in many other locations from the Schengen interior (Bello) to the territory of third countries (Cusumano). Moreover, as well as being spatially and materially diverse, contemporary borderings can also be discursive and/or practice based (Panebianco). As well as being driven by EU level policy and decisionmaking (Kaunert and Leonard), they are produced at and through national level policy discourse (Herborth), practice (Welfens) and societal debate (Futak Campbell).

## Shifting the debate: putting human security at the heart of border security

The starting point of this collection is the fact that ‘[w]hile often understood and policed as static, permanent lines, […] borders are increasingly characterised by movement rather than stasis.’ (Johnson et al. 2011, 65) We are not exploring the relationship between power and space as political geographers do, yet we investigate the politics of shifting borders, migrants’ flows across Mediterranean waves, porous and risky borders crossed by people on the move that EU and EUMS’ public policies cannot guarantee in a safe manner, including offshore control by EU neighbouring states’ coast-guards. Considering ‘sovereign power as a network that moves with and through the bodies of migrants and authorities, never stopping along the edges of sovereign territory, but moving well beyond into grey zones where international and domestic policy, law, and jurisdiction are blurred’ (*idem*), we move beyond a Weberian conception of border that delineates the *locus* of states’ exercise of power.

While not forgetting the important role of the state, we move towards non-state perspectives because ‘bordering is not always the business of the state’ (*idem*: 67) and we are more interested in investigating the broad range of political and societal implications of border security. Exploring the human dimension of border (in)security is the common element of the contributions to this special issue, which acknowledge the plurality of actors involved in securing the borders and critically assess existing border control strategies at EU and EUMS’ level. As they show, migration management—and border making—relies on maritime security provided by navies, coast and board guards but also NGOs; on cooperation practices among state and non-state actors; administrative procedures guaranteeing border security and raft of (often contentious) discursive and ideological underpinnings.

The special issue leverages this work—including through conceptual refinement—to make sense of the implications of the shifting borders of European (in)securities. We look broadly to better understand particular practices and discourses of bordering, and thus of (in)security and (im)mobility, across various locations, but also to zoom out to see their effects on European integration. We thus provide a new and newly comprehensive look at the manifestations of the EU’s migration crisis and its effects on people, states as well as on the EU and Europe more widely. We argue that state security is better achieved via human security and that as long as human security is guaranteed, also state security is guaranteed—assuming an alignment of particular groups of humans with the responsibility of the state. This latter point of course lies at the heart of the EU’s border and migration crisis.

Challenging views that see borders only as sites of state securitisation, we explore humanitarian stances at the European borders. We acknowledge the existing research depicting the ‘false dichotomy’ between state securitisation and humanitarianism, considering the restrictive policies of border and migration control adopted both at national and EU level (Moreno-Lax 2018; Pallister-Wilkins 2018). Yet, we also shed light on neglected *niches* of border security by shifting the border security prism to put the individual at the heart of border security and, thus, enquire into the benefits and politics of ‘human securitization’. Adopting a human-centred perspective, we share the empirical research question: Whose security is at stake? How to guarantee human security in bordering? Depicting pitfalls and shortcomings of EU and EUMS’ discourses and practices on mobility in security, the contributors shed light on European border (in)securities by illuminating the ways that Europeans have enacted divisions between people.

## A guided tour through the contemporary European borderscape

To address ‘Human Security, Border (In)Security and Mobility in Security’, our special issue takes a journey through the European ‘borderscape' (Brambilla et al. 2015). It starts with a conceptual framing that shows how the theoretical and conceptual developments in border studies relate to wider questions of European (in)security and (im)mobility, and which productively links the geographically and thematically diverse contributions that follow (Tallis) and which provides a new lens through which to view the issues at hand. This contribution synthesises and develops recent work on borderscapes to provide a way of making sense of proliferating insecurities and immobilities (Brambilla et al. 2015; Dell’Agnese and Amilhat Szary  2015) and understanding their implications for and in identities and orders. It also urges us to turn critical border studies scholarship to practical, political purpose and provides a framework through which to do so. This contribution conceptually frames the special issue and sensitises us to view the other contributions in both their academic and political contexts.

Thus prepared, we move to the Mediterranean with an examination of humanitarian practices—aimed at reducing the human insecurity of migrants—and their entanglements with Italian practices of border control, primarily driven by the logics of border security (Panebianco). Adopting as case-study the SeaWatch blockade decided in 2019 by the former Italian Minister of the interior, Matteo Salvini, Panebianco explores humanitarian, migrant-centred discourse in the EU periphery and detects via empirical analysis decoupling stances, when and if the issue at stake is to be free from inhuman treatment (Aradau 2004). As well as illuminating core-periphery (geo)politics in the EU, this piece shows the power of humanitarian stigma—in discourse and practice—to put human security at the centre of the conversation on borders.

We then proceed to analyse the discursive and practice-based normalisation of the politics of exception in the Italian migrant reception system (Bello). Focusing on derogatory legal instruments used to normalise the state of exception with indefinite detention and extraordinary measures in the reception system, Bello denounces that ‘insecurity is left to persist across the entire Italian territory’ putting at risk migrants and refugees’ human security. She questions whether Italy can be regarded as a safe country for those in need of a shelter, since migrants in Italy are ‘not in prison but neither free’. This condition of human insecurity is fostered by prejudice (Bello 2017) and calls into question European claims to uphold fundamental rights and freedoms—as well as the EU’s self-declared European values—which in turn questions what kind of order the EU represents.

These cases are then further set in the context of the difficulties and threats that migrants face when trying to cross the Mediterranean and find themselves caught between human smugglers and the ‘walls’ of Fortress Europe. The next contribution looks at how these two poles of action are conditioned by various discourses and practices on (in)security and (im)mobility (Fontana). Exploring smuggling, counter-smuggling, re-smuggling and border-surveillance, Fontana introduces the concept of ‘human insecurity trap’ to grasp the insecurities and vulnerabilities of people on the move. Smuggled to and across Europe, migrants are often entrapped in a spiral of human insecurity at sea, at the state borders and/or across the EU, thus ‘migrants are caught between various ‘rocks’ and ‘hard places’. This leaves them stuck in a series of ‘human insecurity traps’ unfolding at different levels and in different places. Not only does Fontana’s analysis show the fragility of the EU border regime, reflecting ineffective domestic politics at the EU borders (Fontana 2020), it again questions the identity of the EU and its member states as bordering actors and the nature of European order itself.

Moving to the waters off the Libyan coast, the volume then explores the discursive justifications and promotions of anti-migration activism, the parallels it has with those of border control, and its function as a ‘negative image’ of humanitarian and pro-migrant discourses (Cusumano). This contribution on anti-migration activism, driven by ‘identitarian’ groups such as ‘Defend Europe’, provides a clear link between practices in the Mediterranean and discourses of identity politics in EUMS. Drawing on the analysis of the maritime rescue missions conducted by the identitarian youth organisation ‘Defend Europe’, Cusumano demonstrates the existence of institutional isomorphism (Di Maggio and Powell 1983) and discursive frame appropriation that eventually support agendas restricting human mobility. The increasing delegitimisation of sea rescue NGOs has provided an ideal environment for the inception of a new form of right-wing activism that has strategically hijacked some of the discourses and practices developed by humanitarian NGOs.

Another way these discursive politics are expressed is through the categorisation of refugees in European ‘admission programmes’, which again explicitly link the EU interior with externalised border practices and which implicate differing conceptions of security, in facilitating or frustrating practices of mobility. The special issue, the journey, proceeds to look at this aspect of the borderscape with specific regard to German policy and practice in light of attempts to create EU-wide arrangements (Welfens) and their intersections with national policies and debates. Welfens compares the German admissions programme from Lebanon between 2013 and 15 with its arrangements for arrivals from Turkey in 2016. As well as identifying how a more humanitarian approach came to be ‘balanced’ by state security concerns, Welfens suggest that these categorisations of identities and subjectivities, via ‘orders of worth’ end up acting back on the identity of EUMS and of the wider European order.

Staying with Germany, we then explore how countries North of the Alps were forced to ‘become Mediterranean’ in the face of the crisis—at least once it came to their doorsteps in the form of irregular flows of migrants (Herborth). This contribution shows how such instrumental attitudes are rather ‘unbecoming’ for EUMS supposedly committed to ‘European’ solutions on principle and as a result of Schengen obligations and how such an attitude may undermine rather than reinforce Europeanisation and European integration in the long run (Morsut and Kruke 2017; Murray and Longo 2018; Wolf and Ossewaarde 2018). Herborth shows the link between futile attempts to keep migration as a localised problem—and thus to be dealt with by those member states that are geographically Mediterranean—and the futility of ‘European’ solutions to these problems when the polities of EUMS remain locally (nationally) oriented.

Moving to other EUMS that played prominent roles in creating the refugee crisis, the special issue then explores the narrative portrayal of migration in Hungary and Slovakia (Futak-Campbell). The discursive politics in these Visegrad states is shown to be differently configured and has different emphasis than discourse in (e.g.) Germany, but nonetheless to find echoes in and draw from discourses that resound throughout the EU, even if they run contrary to EU policy and (supposedly) desired practice. It shows how different registers of discursive securitisation of migration were employed, ranging from dramatising threats to livelihoods and economic wellbeing, community cohesion and even physical wellbeing of citizens. That this frustrated the formulation and implementation of common and effective EU policy on migration, which could enhance migrants’ human security, as well as the EU and EUMS’ border security, caused some to question how ‘European’ these member states are. It also saw reversion to labelling the Visegrad Four (V4) countries as ‘Eastern European’, even though their discourses and policies were also echoed to greater or lesser degrees elsewhere in the EU. This internal, discursive bordering problematised the EU-European belonging of citizens of these member states and led to calls for them to be excluded from Schengen or even expelled from the Union (see also Tallis, forthcoming). Thus, migration also illuminates other aspects of EU (dis)order and identity.

The special issue ends its journey in Brussels, analysing whether the migration crisis has been or will be used as a window of opportunity for normative and policy change at the EU level, which necessarily implicates national capitals as well (Kaunert and Leonard). This contribution examines the changes, if any, that were triggered by the crisis in the normative frameworks and discourses that underpinned EU policy development on asylum. It maps and assesses the (new) asylum and migration norms and policies that have emerged from or through the crisis and situates this in relation to practices of EU policymaking and in the context of European integration more widely. Kaunert and Leonard look not only at practices and discourses of securitisation—and how they work through ‘association’, in this case of terrorism with migration—but also make a contribution to advancing securitisation theory itself.

Taking this route, from Italy, back and forth across the Mediterranean to Libya and into the EU interior in Germany, Hungary, Slovakia and on to the EU capital, shows the interconnected, ‘cross-border’ nature of European (in)securities relating to migration. It also shows the contingencies and consequences of this borderscape, comprised of related arrays of features, discourses and practices, with regard to questions of European identities and subjectivities, order and governance. We thus collectively shed new light on the significance of the migration crisis for European integration, for people who dwell in Europe and those who wish to.

## Looking ahead: future research paths

Drawing on and developing recent work on borderscapes (e.g. Tallis in this volume and forthcoming) and combining this with insights from (Critical) Security Studies (e.g. Bilgin 2017) and Migration Studies (e.g. Geddes and Scholten 2016), in the context of European (dis)integration (e.g. Schimmelfennig 2021), this collection offers an innovative and unique combination of breadth, depth and coherence in understanding the EU’s migration crisis and its implications on human security at the European borders.

Despite our efforts to include contributions covering a wide spectrum of issues related to European border security, it was not our intention—nor is it the result of this volume—to provide a comprehensive survey of recent literature on borders or related issues. Consequently, there remains much further research to conduct, including by picking up and combining various strands of this research, but also in comparative terms, so to verify the problematisation of border security and its theoretical underpinnings, that we offer in this collection. Bordering in Asia, North America and Australasia are only some of the many sites for comparative fieldwork and, closer to and in Europe, there are myriad aspects of borderings and human insecurities that we do not cover here. Also, the impact of climate change on human mobility in the Global South is drawing increasing research attention (among others, Panebianco 2022). Exploring these sites and themes can deepen our understanding of competing modes of security and mobility at and through borders, our knowledge of transboundary phenomena, moving beyond vulgar applications of securitisation theory to illuminate the impacts of these border politics, practices and discourses on how people can move and dwell as well as the impacts on their lives more broadly, including through the ways they are governed. We hope our collection inspires and supports these endeavours.
